# Growth Performance and Clinicopathological Analyses in Lambs Repetitively Inoculated with Aluminum-Hydroxide Containing Vaccines or Aluminum-Hydroxide Only

**DOI:** 10.3390/ani11010146

**Published:** 2021-01-11

**Authors:** Ricardo de Miguel, Javier Asín, Ana Rodríguez-Largo, Irache Echeverría, Delia Lacasta, Pedro Pinczowski, Marina Gimeno, Jéssica Molín, Antonio Fernández, Ignacio de Blas, Damián de Andrés, Marta Pérez, Ramsés Reina, Lluís Luján

**Affiliations:** 1 Department of Animal Pathology, University of Zaragoza, 50013 Zaragoza, Spain; ricardodemiguel@unizar.es (R.d.M.); jasinros@ucdavis.edu (J.A.); anarlg@unizar.es (A.R.-L.); dlacasta@unizar.es (D.L.); pedro.pinczowski@dpi.nsw.gov.au (P.P.); marina.gimeno@sydney.edu.au (M.G.); jessica.molin@ca.udl.cat (J.M.); afmedica@unizar.es (A.F.); deblas@unizar.es (I.d.B.); mmperez@unizar.es (M.P.); 2 Institute of Agrobiotechnology, CSIC-Government of Navarra, 31192 Mutilva, Spain; irache.echeverria@unavarra.es (I.E.); ancad@unavarra.es (D.d.A.); ramses.reina@unavarra.es (R.R.); 3 Instituto Universitario de Investigación Mixto Agroalimentario de Aragón, University of Zaragoza, 50013 Zaragoza, Spain

**Keywords:** aluminum-hydroxide, aluminum-based adjuvant, aluminum-based vaccine, growth performance, hematology

## Abstract

**Simple Summary:**

Aluminum-hydroxide is an effective vaccine adjuvant used in most commercial sheep vaccines. It facilitates the establishment of a robust immune response against the vaccine antigen. During the first decade of the 21st century, repetitive injections with vaccines containing aluminum-based adjuvants were proposed to be linked to a progressive wasting syndrome in sheep. The aim of this work was to analyze several clinicopathological parameters, including growth performance, clinical data, and histopathological observations in lambs intensively injected with aluminum-containing vaccines, aluminum-hydroxide only, or a saline solution as control. Although aluminum-hydroxide was linked to chronic inflammatory reactions at the injection site and the development of behavioral changes in sheep, the results presented here indicate that injected aluminum-hydroxide, either alone or in combination with vaccine antigens, is not enough to induce relevant changes in the parameters studied. Other factors such as sex, breed, age, production system, diet or climate conditions could play a role in the development of the previously described wasting syndrome.

**Abstract:**

Aluminum (Al) hydroxide is an effective adjuvant used in sheep vaccines. However, Al-adjuvants have been implicated as potential contributors to a severe wasting syndrome in sheep—the so-called ovine autoimmune-inflammatory syndrome induced by adjuvants (ASIA syndrome). This work aimed to characterize the effects of the repetitive injection of Al-hydroxide containing products in lambs. Four flocks (Flocks 1–4; *n* = 21 each) kept under different conditions were studied. Three groups of seven lambs (Vaccine, Adjuvant-only, and Control) were established in each flock. Mild differences in average daily gain and fattening index were observed, indicating a reduced growth performance in Vaccine groups, likely related to short-term episodes of pyrexia and decreased daily intake. Clinical and hematological parameters remained within normal limits. Histology showed no significant differences between groups, although there was a tendency to present a higher frequency of hyperchromatic, shrunken neurons in the lumbar spinal cord in the Adjuvant-only group. Although Al-hydroxide was linked to granulomas at the injection site and behavioral changes in sheep, the results of the present experimental work indicate that injected Al-hydroxide is not enough to fully reproduce the wasting presentation of the ASIA syndrome. Other factors such as sex, breed, age, production system, diet or climate conditions could play a role.

## 1. Introduction

Vaccines are indispensable tools in animal production to control diseases and increase production rates [1]. In sheep husbandry, vaccination protocols differ depending on a variety of factors such as the production system, geographical location, climate, and/or disease prevalence [2]. Furthermore, health management programs can be modified by compulsory vaccination campaigns to fight against emerging or re-emerging epizootics [3]. A recent example was the compulsory vaccination campaign against bluetongue virus that took place in most European countries during the first decade of the 21st century [4,5]. This immunization campaign effectively controlled virus circulation and stopped disease progression. However, the repetitive vaccination caused diverse side effects of variable intensity that affected productive parameters and animal health in several countries [6,7,8,9,10]. Interestingly, a wasting syndrome associated with neurological signs was described and the aluminum (Al)-based adjuvants—that the used vaccines contained—were incriminated as the potential triggering etiology [11]. The name ovine autoimmune/inflammatory syndrome induced by adjuvants (ASIA syndrome) was proposed for this process [11,12].

In veterinary medicine, Al-hydroxide is a widely employed vaccine adjuvant that efficiently boosts immune responses against the vaccine antigens [13,14]. Therefore, Al is currently present in most ovine commercial vaccines. Previous publications demonstrated that subcutaneous inoculation of Al-hydroxide adjuvants induces the formation of persistent, sterile granulomas composed of abundant Al-laden macrophages in the experimental animals used in the present study [15]. These macrophages can reach regional lymph nodes and potentially disseminate Al throughout the body [15]. Indeed, higher Al levels were demonstrated in the lumbar spinal cord of the Al-hydroxide-inoculated animals [16]. Moreover, Al-hydroxide was linked to the development of an array of behavioral changes in a group of the same lambs [17]. The evaluation of productive and clinical parameters together with a comprehensive pathological analysis in the animals included in the aforementioned publications have never been reported. Moreover, whether repetitive inoculation of Al-hydroxide may induce an ovine wasting syndrome or not is a crucial question that has never been addressed in a large-scale experiment.

The aim of this work was to study the clinical long-term effects and postmortem changes induced by the repetitive injection of Al-hydroxide, either alone or combined into commercial vaccines, in lambs maintained under different environmental conditions and productive systems.

## 2. Materials and Methods

### 2.1. Experimental Design

All procedures were carried out under Project License PI15/14 approved by the Ethics Committee for Animal Experiments of the University of Zaragoza. The care and use of animals were performed according to the Spanish Policy for Animal Protection RD53/2013, which meets the European Union Directive 2010/63 on the protection of animals used for scientific purposes.

A total of 84, three-month-old, neutered male lambs were divided into four flocks of 21 animals each. Flock 1 originated from a Rasa Aragonesa breed-accredited commercial farm and was placed in a research facility (Experimental farm, University of Zaragoza) under previously described conditions [15,17]. Animals from flocks 2, 3, and 4 were born, selected, and raised in commercial sheep farms located in different geographical areas [14]. Flocks 2, 3, and 4 remained integrated in their original herd for the entire duration of the experiment. Detailed information of the production systems and climatological parameters is provided in Table 1 and Table A1 (Appendix A), respectively.

Each flock of 21 lambs was split into three treatment groups of 7 animals each: Vaccine group, which was inoculated with commercial vaccines; Adjuvant-only group, which received the equivalent dose of Al-hydroxide (Alhydrogel^®^, CZ Veterinaria, Porriño, Spain), and Control group, which was injected with phosphate-buffered saline (PBS). Six animals (i–vi) died for reasons unrelated to the treatments: in Flock 3, these included two animals in the Control group (i: urolithiasis and hydronephrosis; ii: aspiration pneumonia), one animal in the Vaccine group (iii: urolithiasis and hydronephrosis), and one animal in the Adjuvant-only group (iv: urolithiasis and hydronephrosis); in Flock 4, dead animals included one animal in the Adjuvant-only group (v: septicemia caused by *Pasteurella* spp.) and one animal in the Vaccine group (vi: sheep bloat). The final number of animals in each flock was: Flock 1: *n* = 21; Flock 2: *n* = 21; Flock 3: *n* = 17; and Flock 4: *n* = 19. Therefore, when all flocks were grouped together, each treatment group (Vaccine, Adjuvant-only, Control) consisted of 26 animals at the end of the experiment. Data derived from dead animals were not considered for any of the parameters evaluated.

**Figure 1:**
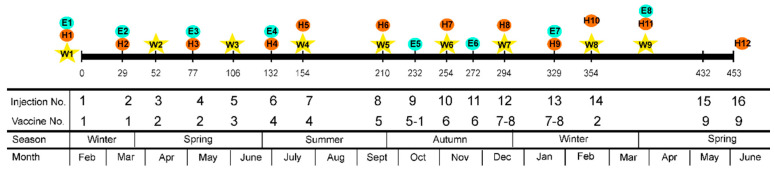
Global inoculation schedule. Each injection date is indicated by a vertical line and a number (mean value of dpi of the four flocks). W: Weight measurement. E: Clinical examination. H: Hematological analysis. Information on the injection and vaccines number, season, and month is also provided. Inoculation schedule for each individual flock is provided in Figure A1 (Appendix B). Information about the vaccines used is presented in Table A2 (Appendix C).

**Figure A1:**
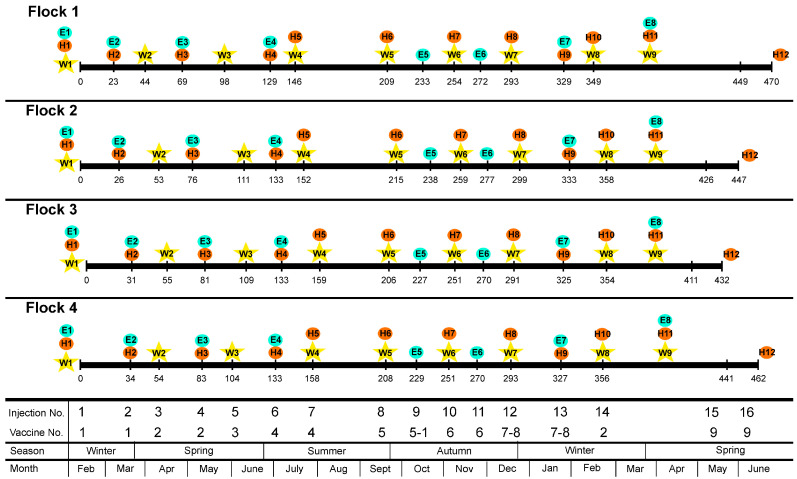
Inoculation schedule for each flock individually. All flocks were subjected to the same inoculation schedule and experimental procedures. Differences in the number of days between inoculations in the different flocks and other experimental procedures are shown. Each injection date is indicated by a vertical line and a number. W: Weight measurement. E: Clinical examination. H: Hematological analysis. Information on the injection and vaccines number, season, and month is also provided. Information about the vaccines used is presented in Table A2 (Appendix C).

An accelerated vaccination schedule was applied. The goal was to reproduce, within an acceptable time frame for a 3-year research project, the management field conditions that led to the ovine ASIA syndrome. Animals received a total of 19 subcutaneous inoculations, which mimic the amount of Al that animals can receive during their productive lifespan (a mean of seven years). The last injection was applied 5 days prior to euthanasia in the four flocks. Inoculation schedule is described in Figure 1 and Figure A1 (Appendix B). Details of the vaccines used are described in Table A2 (Appendix C). Vaccine and Adjuvant-only groups received a total of 81.29 mg of Al. The study lasted 15 months, ranging from 432 to 470 days, depending on each flock.

### 2.2. Productive and Clinical Parameters

In order to analyze animal growth, lamb weights were recorded nine times along the experiment, days between each measurement ranged from 31 to 63 (Figure 1, W1 to W9). Partial and global average daily gain (ADG) were calculated. Partial ADG included all the weighing dates; global ADG was calculated using the first and the last weights and dividing the difference by the number of days between them. General clinical examination was performed periodically (Figure 1), 18 to 41 days after previous inoculation date and just prior to the application of the next inoculation. It included blood sampling, rectal temperature, heart rate, and respiratory rate. Blood samples were obtained by jugular venipuncture with 6 mL EDTA tubes (BD Vacutainer^®^, Becton Dickinson, Madrid, Spain) and a hematological panel including white blood cell count, red blood cell count, hematocrit, hemoglobin, and platelet count was performed (scil Vet abc Plus™ Animal Blood Counter). Additionally, animals from Flock 1 were subjected to two rounds of behavioral tests (one in summer and another in winter) and these results were previously reported [17]. Urine was analyzed just after euthanasia with a biochemical strip to test pH, glucose, and protein.

### 2.3. Post-Mortem Studies

Euthanasia was performed by intravenous injection of an overdose of barbiturate solution (Dolethal^®^, Vetoquinol, Madrid, Spain). Complete post-mortem examinations were performed. Perirenal, mesenteric, pericardial, thoracic, and subcutaneous fat deposits were scored from 0–3 (0: Absence of fat; 1: Scarce fat deposition; 2: Moderate fat deposition; 3: Normal fat deposition), and a fattening index was calculated as the mean value of these five scores. Additionally, thickness of subcutaneous sternal fat was measured.

Systematic sampling of all tissues was performed. Central nervous system (CNS) and peripheral nervous system (PNS) were sampled following a previously-described protocol [18]. Tissues were fixed in 10% neutral-buffered formalin for 48–72 h. Samples were routinely processed for paraffin embedding and production of 4 µm, hematoxylin-eosin (HE)-stained slides. Histopathological analysis of different areas of the CNS (brain: frontal cortex-caudate nucleus, parietal cortex, thalamus-hypothalamus; spinal cord: cervical, thoracic, and lumbar segments), PNS (subcutaneous-thoracic, sciatic, tibial, and radial nerves), liver, kidney, pancreas, spleen, adrenal glands, thyroid, and thymus were performed by a single pathologist (J.A.) who was blinded to the treatment group. The histopathological features evaluated, and the scoring system used in each tissue are described in Table A3, Table A4, Table A5, Table A6, Table A7, Table A8, Table A9, Table A10 and Table A11 (Appendix D).

### 2.4. Statistical Analysis

All statistical analyses were performed using IBM SPSS 19.0 for Windows (IBM Corp., Armonk, NY, USA). Quantitative variables (i.e., body weight, ADG, fattening index, sternal fat deposits) were analyzed by Shapiro–Wilk test to assess normality of data. Levene’s test was used to test the equality of variances. When data followed a normal distribution and had homogeneous variances, the parametric test ANOVA was used, followed by Duncan’s multiple range test as a *post hoc.* In normally-distributed quantitative variables with unequal variances, Welch’s t-test was used. In non-normal quantitative variables, the non-parametric Kruskal–Wallis test was used, followed by Dunn’s test as a *post hoc*. In qualitative variables (i.e., histopathological analyses), assessment of the association between groups was carried out using Pearson’s chi-square test or alternatively Likelihood ratio test and Fisher’s exact test when needed. Statistical significance was considered when *p* value < 0.05. Statistical tendency was considered when *p* value ≤ 0.1.

## 3. Results and Discussion

### 3.1. Body Weight and Average Daily Gain

**Figure 2:**
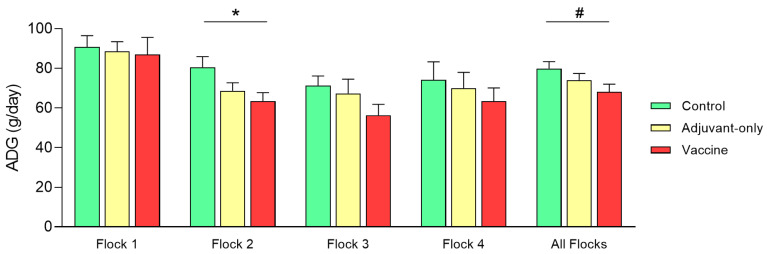
Global average daily gain (ADG) along the experiment in Control (green), Adjuvant-only (yellow), and Vaccine groups (red), both in each individual flock and in all flocks grouped together (All Flocks). Data represented as mean and Standard Error. *: statistical significance (*p* < 0.05); #: statistical tendency (*p* ≤ 0.1).

Results for body weight and ADG are presented in Table A12 (Appendix E) and Table A13 (Appendix F), respectively. Mild to moderate differences in ADG were observed between treatment groups in each one of the individual flocks. Global ADG of each flock is represented in Figure 2 and indicated a moderate growth rate reduction in Vaccine groups in contrast with Control groups. Adjuvant-only groups showed lower ADG values than Control groups but higher ADG values than Vaccine groups. This data distribution was observed for the ADG values of all flocks, although Flock 2 was the only one where these differences were statistically significant (*p* = 0.045). Moreover, when all flocks were grouped together, this tendency was maintained although it did not reach significance (*p* = 0.072).

This lower ADG for the Vaccine and—to a lesser extent—Adjuvant-only groups could be explained by transient, short-term, post-vaccination events, including brief periods (24–48 h) of fever after vaccinations and associated decreased appetite [19,20]. Indeed, it has been observed that booster vaccinations against respiratory pathogens in fattening lambs can cause moderate growth retardation, with animals reaching their optimal sacrifice weight 5 days later than control animals (JM Gonzalez, personal communication). The lambs included in this work likely suffered repetitive episodes of hyperthermia and decreased daily intake, which could have affected ADG and absolute weight at the end of the experiment. In fact, the acute-phase response elicited by vaccination is essential for optimal development of the immune response [21,22]. This response increases nutrient demands so they are redistributed to support the immune system instead of growing, which may lead to reduced growth performance and feed efficiency [23,24]. Moreover, stimulation of immune response can activate the mammalian target of rapamycin (mTOR) signaling pathway and thus affect metabolic routes involved in reduced anabolism [25,26]. The latter is in accordance with energy consumption due to vaccination and may affect the body condition in specific vaccination strategies, especially in negatively energy balanced feedlot animals. In such a scenario, the presence of more severe inflammatory reactions in the injection sites of animals in the Vaccine groups [15] might also help to explain the differences between Vaccine and Adjuvant-only groups. None of the lambs injected with the adjuvant only or with Al-containing vaccines unequivocally developed a wasting syndrome such as the one described after the compulsory vaccination campaigns against bluetongue [11].

Analysis of partial variations in ADG revealed significant differences between weight measurements at dates W4 and W5 (Table A13—Appendix F), coinciding with the summer (Figure 1). In Flocks 1 and 2, Vaccine groups showed a significantly lower ADG than Control and Adjuvant-only groups (Flock 1: *p* = 0.02; Flock 2: *p* = 0.049). Flock 4 showed similar, although non-significant (*p* = 0.055) results. No statistically significant variation was observed in Flock 3. When the four flocks were considered altogether, these variations in the Vaccine group also reached statistical significance (*p* = 0.045). Globally, these variations in ADG are likely associated with the high temperatures reached during this period and detailed in Table A1 (Appendix A). High environmental temperatures induce heat stress and negatively alter lamb growth due to lower feed intake and activation of thermoregulatory mechanisms [27]. Thermoregulatory capacity and productive performance in fattening lambs with heat stress depends on breed, production system, diet, and age [28]. Perhaps these effects were more marked in the Vaccine group because they combined with preexisting stressors in these animals, i.e., persistent injection site reactions [15]. Interestingly, transcriptomic studies performed in Flock 1 of the present work demonstrated that Al adjuvants significantly increased the expression of pro-inflammatory cytokines and genes of the NF-kB and apoptotic pathways [29]. Activation of these pathways may potentially interfere with optimal thermoregulatory mechanisms.

### 3.2. Clinical and Hematological Examination

Rectal temperatures, heart and respiratory rates, and urine analyses showed no relevant differences between groups in any of the flocks individually or when all flocks were grouped together. Transient pyrexia is a common and expectable post-vaccination effect in feedlot lambs and calves, especially after booster vaccinations [19,20]. In our study, rectal temperature was recorded 18 to 41 days after the previous inoculations (Figure 1), as the main objective was to measure the cumulative, long-term effect of the repetitive injections rather than short-term variations. In this context, it is likely that those transient differences were missed.

Hematological results of the three treatment groups of the four flocks grouped together are detailed in Table A14 (Appendix G). There were point differences between groups both at the individual flock level and when all flocks were considered together, but data were always within normal ranges for sheep. Marked normochromic, non-regenerative anemia was reported as part of the wasting syndrome described after the compulsory bluetongue vaccination campaign [11], but this phenomenon was not observed in this experimental work. This might be due to different factors influencing the development of that particular feature, as experimental conditions in the present study probably could not reproduce the exact scenario that fueled the appearance of the wasting presentation of the ovine ASIA syndrome.

### 3.3. Post-Mortem Studies

Necropsy findings revealed mild differences in the fattening index and sternal fat deposits (Table 2) when all flocks were considered together. For both parameters, Vaccine group showed lower values than Control group, whereas values in the Adjuvant-only group were higher than the Vaccine group and lower than the Control group. These results parallel the mild differences observed in the ADG of these animals. Therefore, decreased fat deposition at the end of the experiment in the Vaccine group may be also the result of transient periods of anorexia. Sternal fat deposits play an important role in thermogenesis in sheep [30]. There were no other gross abnormalities in any of the treatment groups apart from those previously described [15].

Histopathological results of the four flocks grouped together are detailed in Table 3 and Table A15 (Appendix H). Evaluation of the CNS and PNS showed point differences between treatment groups when each flock was analyzed individually, but they were heterogeneous between flocks and not clearly linked to treatments applied. However, when all flocks where grouped together only a statistical tendency (*p* = 0.100) to present higher numbers of dark neurons in the lumbar spinal cord (Table 3) was observed in the Adjuvant-only group. The term “dark neuron” defines a hyperchromatic, shrunken neuron [31,32]. This histological finding should be interpreted cautiously as it may be just an artifact [32]. Degenerated necrotic neurons tend to be brightly acidophilic rather than basophilic/dark, although sometimes these two appearances are difficult to differentiate. Furthermore, ischemic neurons in peracute stages of degeneration may be indistinguishable from dark neurons [33,34]. Interestingly, analytical measurements and a lumogallion stain (Al-specific histochemical stain) performed in the CNS of animals from Flock 1 revealed increased levels of Al in the lumbar spinal cord of the Adjuvant-only group [16]. Perhaps this tendency in the number of dark neurons in the spinal cord of the Adjuvant-only group is related to Al accumulation in the same location. Remarkably, this global absence of histological lesions in the encephalon was observed in animals from Flock 1, which showed significant behavioral alterations in a previous study [17]. Furthermore, transcriptomic studies performed in the encephalon of these animals revealed dysregulation of genes related to neurological function and mitochondrial energy metabolism [35]. Most likely, these clinical and molecular differences did not induce structural abnormalities that could be detected with basic histological methods such as HE.

The pancreas showed a significantly (*p* = 0.012) increased presence of multifocal and/or periductal lymphoplasmacytic inflammatory infiltrates in the Adjuvant-only group when all flocks were considered together (Table 4). Interestingly, pancreatic changes have been reported in guinea pigs inoculated with Al-hydroxide adjuvants either subcutaneously or intraperitoneally [36]. Histopathological results obtained in the rest of organs are presented in Table A16, Table A17, Table A18, Table A19, Table A20 and Table A21 (Appendix I). There was a positive tendency (*p* = 0.078) in the number of lambs with thyroid follicular cell hypertrophy in the Adjuvant-only and Vaccine groups (Table A20—Appendix I), and a significant (*p* = 0.043) decrease in the number of lambs showing thymic germinal center hyperplasia in the Adjuvant-only and Vaccine groups (Table A21—Appendix I). No significant differences were found in any of the parameters analyzed in liver, kidney, spleen, and adrenal gland.

### 3.4. Study Limitations

The interpretation of these results has some limitations intrinsic to the study design and experimental procedures performed. First, the number of animals used could have limited some of the statistical analyses. Second, most of the descriptions of the wasting syndrome that occurred after the bluetongue vaccination campaigns included adult animals, generally ewes in full production [11]. The animals used in this experiment were growing, male neutered, young lambs, which perhaps limited the capacity of the inoculations to induce severe weight loss. A similar study using adult sheep with stable body weight at the beginning of the experiment could help to clarify this aspect. Lastly, the number of inoculations performed overrates the normal vaccination schedule for sheep in a year. In fact, the wasting syndrome occurred with just four doses in around a month, with an amount of 16 mg of Al inoculated per animal [10,11]. Most likely, in addition to Al, other parameters such as sex, breed, age, productive system, diet, and/or climate conditions (winter cold) are necessary co-factors for the full development of the devastating wasting presentation of the ovine ASIA syndrome.

## 4. Conclusions

This work summarizes the results obtained on the growth performance and clinicopathological parameters in lambs subjected to repetitive inoculations with saline solution (Control group), Al-hydroxide adjuvants (Adjuvant-only group) or Al-hydroxide-based vaccines (Vaccine group) either under experimental or in field conditions. Mild differences in ADG and fattening index were reported in the Vaccine group and were likely associated with transient post-injection hyperthermia with decreased daily intake and/or intense inflammatory reactions occurring at the injection sites [15]. Clinical, hematological, and histopathological analyses revealed minimal abnormalities, even knowing that previous behavioral and transcriptomic studies performed in one of the flocks studied here revealed significant alterations in the Adjuvant-only and/or Vaccine groups [17,35]. Despite previously-observed results showing the effects of repetitive inoculations of Al-hydroxide containing vaccines and adjuvants in sheep [15,16,17,29,35], the results or this experimental study seem to indicate that injected Al may be necessary, but not sufficient to reproduce all the productive and clinicopathological characteristics of the ovine wasting syndrome (ovine ASIA syndrome) [11].

## Figures and Tables

**Table 1:** Characteristics of the lambs and flocks used in the experiment.

Flock	Breed	Management	Shepherding
1	Rasa Aragonesa purebred	Experimental farm	No
2	Rasa Aragonesa × Romanov crossbred	Intensive	No
3	Rasa Aragonesa × Romanov crossbred	Extensive	Yes
4	Rasa Aragonesa purebred	Extensive	Yes

**Table 2:** Fattening index and sternal fat deposits in Control, Adjuvant-only, and Vaccine groups (*n* = 26 each) when all flocks were considered together. Data represented as mean, standard deviation (SD), and interquartile rank (IQR).

Group	Fattening Index				Sternal Fat Deposits				
	Mean	SD	IQR	*p*		Mean	SD	IQR	*p*
Control	2.83	0.17	2.80–3.00 ^a^			3.74	0.38	3.50–4.00 ^a^	
Adjuvant-only	2.71	0.31	2.60–3.00 ^a^			3.58	0.70	3.00–4.27 ^ab^	
Vaccine	2.52	0.38	2.30–2.80 ^b^	0.003 ^KW^*		3.32	0.52	3.00–3.50 ^b^	0.008 ^KW^*

^a,b^: Statistically significant differences between groups based on *post hoc* test. ^KW^: Kruskal–Wallis test. *: Statistically significant (*p* < 0.05).

**Table 3:** Histopathological findings in the central nervous system in Control, Adjuvant-only (Adjuvant), and Vaccine groups (*n* = 26 each) of all flocks grouped together. Data provided as animals with the referred histological lesion relative to the total number of animals analyzed. Methodology of histopathological evaluation is detailed in Table A3, Table A4, Table A5, Table A6, Table A7, Table A8, Table A9, Table A10 and Table A11 (Appendix D).

Location	Group	Perivascular Cuffing	Meningitis	Glial Nodules	Microglial Activation	Dark Neurons
Frontal cortex and Caudate nucleus	Control	8/26	0/26	19/26	6/26	22/26
	Adjuvant	10/26	2/26	19/26	4/26	23/25
	Vaccine	7/26	2/26	14/26	2/26	22/26
	*p*	0.662 ^Xi^	0.187 ^LR^	0.236 ^Xi^	0.239 ^LR^	0.645 ^LR^
Parietal cortex	Control	7/26	1/26	3/26	6/26	21/26
	Adjuvant	6/26	2/26	2/26	4/26	22/26
	Vaccine	2/26	2/26	2/26	3/26	22/26
	*p*	0.177 ^Xi^	0.808 ^Xi^	0.859 ^LR^	0.528 ^LR^	0.913 ^LR^
Thalamus and Hippothalamus	Control	8/26	0/26	3/26	7/26	24/26
	Adjuvant	4/26	0/26	1/26	12/26	25/26
	Vaccine	7/26	1/26	4/26	11/26	24/26
	*p*	0.495 ^Xi^	0.329 ^LR^	0.335 ^LR^	0.311 ^LR^	0.793 ^LR^
Cervical spinal cord	Control	3/26	2/26	1/26	0/26	9/26
	Adjuvant	2/26	1/26	0/26	0/26	12/26
	Vaccine	1/26	0/26	0/26	0/26	11/26
	*p*	0.568 ^LR^	0.240 ^LR^	0.329 ^LR^	-	0.690 ^Xi^
Thoracic spinal cord	Control	0/26	0/26	0/26	0/26	17/26
	Adjuvant	1/26	0/26	0/26	0/26	16/26
	Vaccine	0/26	0/26	0/26	0/26	10/26
	*p*	0.329 ^LR^	-	-	-	0.108 ^Xi^
Lumbar spinal cord	Control	1/26	0/26	0/26	24/26	13/26
	Adjuvant	1/26	0/26	1/26	25/26	20/26
	Vaccine	0/26	0/26	0/26	24/26	14/26
	*p*	0.439 ^LR^	-	0.329 ^LR^	0.793 ^LR^	**0.100 ^Xi#^**

^Xi^: Pearson’s chi square test. ^LR^: Likelihood ratio test. ^#^: Statistical tendency (*p* ≤ 0.1).

**Table 4:** Inflammation (i.e., interstitial and/or periductal aggregates of lymphocytes, plasma cells, and/or histiocytes) in the pancreas in Control, Adjuvant-only (Adjuvant), and Vaccine groups (*n* = 26 each) of all flocks grouped together. Data provided as animals with the histological lesion relative to the total number of animals analyzed. Methodology of histopathological evaluation detailed in Table A3, Table A4, Table A5, Table A6, Table A7, Table A8, Table A9, Table A10 and Table A11 (Appendix D).

	Control	Adjuvant	Vaccine	*p*
Inflammation	1/26	8/26	2/26	0.012 ^LR^*

^LR^: Likelihood ratio test. *: Statistical significance (*p* < 0.05).

## Data Availability

Our study includes all data as Appendix A, Appendix B, Appendix C, Appendix D, Appendix E, Appendix F, Appendix G, Appendix H and Appendix I.

## Appendix A

**Table A1:** Climate conditions along the experiment. Higher and lower temperatures during the experiment are in bold. Higher relative humidity along the experiment is indicated in bold. Data obtained from the State Meteorological Agency (AEMET) of the Spanish Government [37].

	FLOCK 1 and 4								FLOCK 2								FLOCK 3							
Month–Year	T. mean ^3^	T. min ^1^		T. max ^2^		N0 ^4^	N30 ^5^	RH ^6^	T. mean ^3^	T. min ^1^		T. max ^2^		N0 ^4^	N30 ^5^	RH ^6^	T. mean ^3^	T. min ^1^		T. max ^2^		N0 ^4^	N30 ^5^	RH ^6^
		Mean	Abs	Mean	Abs					Mean	Abs	Mean	Abs					Mean	Abs	Mean	Abs			
January–2015	**7.1**	2.5	−2.0	11.6	16.7	**7**	0	66	**6.0**	**1.1**	−**5.6**	10.8	16.9	**14**	0	N/A ^7^	**5.9**	**1**	−1.8	10.7	17.5	**10**	0	75
February–2015	**7.1**	**2.8**	−**2.9**	11.3	18.4	**7**	0	61	6.9	2.1	−4.9	11.8	18.4	10	0	N/A	6.3	1.2	−**5.2**	11.4	16.7	8	0	65
March–2015	11.8	6.7	1.3	16.9	24.0	0	0	56	11.6	6.1	−1.1	17.1	23.7	3	0	N/A	11.4	5.6	0.1	17.1	23.0	0	0	61
April–2015	15.6	9.4	4.6	21.8	27.9	0	0	46	14.5	7.6	1.3	21.4	26.8	0	0	54	14.5	7.9	2.4	20.9	25.5	0	0	52
May–2015	20.1	13.5	9.4	26.5	36.4	0	9	43	19.1	11.1	4.6	27	34.0	0	7	47	18.9	11.2	5.0	26.6	35.1	0	6	43
June–2015	25.2	17.5	14.0	32.9	41.6	0	20	38	23.4	15.2	11.7	31.6	39.1	0	20	50	23.4	15.6	10.6	31.2	38.6	0	19	44
July–2015	**27.9**	20.2	16.2	**35.5**	**43.7**	0	**27**	38	**26.7**	18.5	13.3	**34.7**	**42.8**	0	**28**	48	**27.3**	19.1	12.5	**35.5**	**42.1**	0	**29**	39
August–2015	25.5	18.8	14.2	32.1	37.2	0	24	45	24.2	17.3	11.0	31.1	36.8	0	21	58	24.2	17.2	11.8	31.3	36.5	0	21	49
September–2015	20.5	14.9	10.7	26.1	30.4	0	2	48	19.1	12.8	6.8	25.4	30.2	0	1	59	19	12.8	8.5	25.1	30.1	0	2	59
October–2015	16.6	11.5	4.9	21.7	28.3	0	0	58	15.4	9.6	2.3	21.2	27.4	0	0	66	15.8	10.3	2.7	21.3	26.3	0	0	66
November–2015	12.2	8	1.7	16.4	24.8	0	0	73	10.9	7	−3.6	14.8	22.1	3	0	80	10.9	7.2	−0.9	14.6	23.8	1	0	81
December–2015	7.6	3.9	−0.2	11.2	16.6	1	0	**82**	7.3	3.4	−1.0	11.1	16.4	4	0	**87**	8.6	4.7	−0.9	12.5	17.9	3	0	**84**
January–2016	9.6	5.9	0.2	13.3	20.5	0	0	70	7.8	3.4	−2.1	12.2	18.4	4	0	78	7.8	4.1	−1.7	11.5	16.5	2	0	81
February–2016	9.5	4.7	-0.8	14.2	21.2	2	0	60	8	2.5	−4.1	13.5	19.6	9	0	70	8.2	3.2	−3.9	13.1	18.7	4	0	71
March–2016	10.3	5.5	0.7	15.1	24.9	0	0	58	9.2	3.5	−1.9	14.8	24.2	2	0	67	9.3	3.6	−1.3	15.1	22.4	2	0	66
April–2016	14	8.5	2.6	19.4	26.9	0	0	51	12.8	6.4	1.0	19.3	26.5	0	0	60	12.3	6.2	1.5	18.4	23.8	0	0	62
May–2016	17.9	12.1	6.8	23.7	31.3	0	2	48	16.4	9.4	2.2	23.3	29.5	0	0	57	15.8	9.1	1.2	22.5	30.2	0	1	57
June–2016	23.4	16.4	11.3	30.3	37.0	0	17	40	22.2	14.2	8.4	30.1	34.9	0	16	47	21.7	13.9	7.7	29.5	35.9	0	14	42

^1^ T. min: Minimum temperature (Mean: Mean of the minimum temperature/Abs: Lowest value for a specific month). ^2^ T. max: Maximum temperature (Mean: Mean of the maximum temperature/Abs: Highest value for a specific month). ^3^ T. mean: Mean temperature for a specific month. ^4^ N0: Number of days with the minimum temperature under 0 °C. ^5^ N30: Number of days with the maximum temperature over 30 °C. ^6^ RH: Relative humidity. ^7^ N/A: Not available.

## Appendix C

**Table A2:** Vaccines used in the experiment and inoculation date. Aluminum (Al) content was established by inductively coupled mass spectrometry (ICP-MS) and calculated as milligrams (mg) per total dose.

Vaccine Number	Commercial Name	Antigen/s	Inoculation Date (Figure 1)	Al per Dose (mg)
1	Heptavac P Plus	*Pasteurella multocida* *Mannheimia haemolytica* *Clostridium* spp.	1, 2, 9	7.5
2	Autogenous vac.	*Staphylococcus aureus* spp. *anaerobius*	3, 4, 14	1.644
3	Vanguard R	Rabies virus	5	1.025
4	Agalaxipra	*Mycoplasma agalactiae*	6, 7	6.764
5	Ovivac CS	*Chlamydia abortus* *Salmonella abortus ovis*	8, 9	5.6
6	Autogenous vac.	*Corynebacterium pseudotuberculosis*	10, 11	1.32
7	Bluevac-1	Bluetongue virus Serotype 1	12, 13	4.18
8	Bluevac-4	Bluetongue virus Serotype 4	12, 13	4.16
9	Bluevac BTV8	Bluetongue virus Serotype 8	15, 16	4.4

## Appendix D

Histopathological features evaluated in the experimental lambs in central and peripheral nervous systems, liver, kidney, pancreas, spleen, adrenal glands, thyroid, and thymus.

**Table A3:** Histopathological features evaluated in the central nervous system (brain: frontal cortex-caudate nucleus, parietal cortex, thalamus-hypothalamus; spinal cord: cervical spinal cord, thoracic spinal cord, lumbar spinal cord).

Features	Evaluation	Description
Perivascular cuffing	P/A ^1^	At least one blood vessel surrounded by >2 layers-thick perivascular cuff of lymphocytes, plasma cells, and/or histiocytes.
Meningitis	P/A	Aggregates of lymphocytes, plasma cells, and/or histiocytes in the meninges
Glial nodules	P/A	At least one nodular aggregate of glial cells in the neuropil
Microglial activation	P/A	Aggregates of rod shaped glial cells in the neuropil
Dark neurons	P/A	Deeply hyperchromatic, shrunken neurons

^1^ P/A: Presence/Absence.

**Table A4:** Histopathological features evaluated in the peripheral nervous system (subcutaneous-thoracic, sciatic, tibial, and radial nerves).

Features	Evaluation	Description
Perineural, perivascular cuffing	P/A ^1^	At least one, ≥1 layer thick, perivascular aggregate of lymphocytes, plasma cells, and/or histiocytes in the tissues adjacent to the nerve
Intraneural inflammation	P/A	Aggregates of lymphocytes, plasma cells, and/or histiocytes within the peri- or endoneurium

^1^ P/A: Presence/Absence.

**Table A5:** Histopathological features evaluated in the liver.

Features	Evaluation	Description
Portal/periportal inflammation	P/A ^1^	Inflammatory infiltrates in or around portal spaces
	Type	LP: Lymphoplasmacytic LP + E: Lymphoplasmacytic and eosinophilic
Hepatocellular degeneration	P/A	Swollen hepatocytes with vacuolated or feathery cytoplasm
Hepatocellular necrosis	P/A	Shrunken eosinophilic hepatocytes with pyknotic nucleus
Hepatocellular atrophy	P/A	Shrunken hepatocyte cords with distended sinusoids

^1^ P/A: Presence/Absence.

**Table A6:** Histopathological features evaluated in the kidney.

Features	Evaluation	Description
Glomeruli: Proteinuria	P/A ^1^	Protein globules in the Bowman’s space
Tubules: Degeneration	P/A	Swollen tubular epithelium with vacuolated or feathery cytoplasm
Tubules: Hyaline droplets	P/A	Deeply eosinophilic, 1–3 μm intracytoplasmic droplets
Interstitium: Inflammation	P/A	Aggregates of lymphocytes, plasma cells, and/or histiocytes
Medulla: Mineralization	P/A	Foci of tubulointerstitial mineralization

^1^ P/A: Presence/Absence.

**Table A7:** Histopathological features evaluated in the pancreas.

Features	Evaluation	Description
Inflammation	P/A ^1^	Interstitial and/or periductal aggregates of lymphocytes, plasma cells, and/or histiocytes

^1^ P/A: Presence/Absence.

**Table A8:** Histopathological features evaluated in the spleen.

Features	Evaluation	Description
White pulp hyperplasia	P/A ^1^	Prominent lymphoid follicles with increased numbers of lymphocytes/blasts
Perifollicular PMs ^2^	P/A	Aggregates of neutrophils and/or eosinophils around the lymphoid follicles

^1^ P/A: Presence/Absence. ^2^ PMs: Polymorphonuclear leukocytes.

**Table A9:** Histopathological features evaluated in the adrenal gland.

Features	Evaluation	Description
Cortical hyperplasia	P/A ^1^	Thickened adrenal cortex
	Localization	Fascicular Reticular Both
Cortical inflammation	P/A	Aggregates of lymphocytes, plasma cells, histiocytes, and/or neutrophils in the cortex

^1^ P/A: Presence/absence.

**Table A10:** Histopathological features evaluated in the thyroid gland.

Features	Evaluation	Description
Inflammation	P/A ^1^	Aggregates of lymphocytes, plasma cells, and/or histiocytes in the interstitium
Follicular cells hyperplasia	P/A	Increased numbers of follicular cells
Follicular cells hypertrophy	P/A	Increased size of follicular cells
C cells hyperplasia/hypertrophy	P/A	Increased number and/or size of C cells

^1^ P/A: Presence/absence.

**Table A11:** Histopathological features evaluated in the thymus.

Features	Evaluation	Description
Germinal centers	P/A ^1^	Presence of conspicuous germinal centers in >80% of the follicles
Degree of involution	0	No involution: Well-formed follicles.
	1	Mild involution: Smaller follicles.
	2	Moderate involution: Smaller follicles with fat-filled areas between them.
	3	Severe/total involution: Rare thymic remnants

^1^ P/A: Presence/absence.

## Appendix E

**Table A12:** Body weight (W) along the experiment in Control, Adjuvant-only, and Vaccine groups in each of the four flocks individually (Flock 1–4) and all flocks grouped together (All Flocks). Data represented as mean and standard deviation (SD).

	Group	Flock 1			Flock 2			Flock 3			Flock 4			All Flocks		
		*n* = 21			*n* = 21			*n* = 17			*n* = 19			*n* = 78		
		Mean	SD	*p*	Mean	SD	*p*	Mean	SD	*p*	Mean	SD	*p*	Mean	SD	*p*
W1	Control	31.68	3.7	0.942 ^KW^	38.26	3.4	0.381 ^A^	38.30	2.7	0.956 ^A^	38.61	2.2	0.857 ^A^	36.59	4.2	0.611 ^A^
	Adjuvant	31.28	4.6		37.71	4.4		38.08	3.4		38.03	2.6		36.14	4.7	
	Vaccine	31.83	3.4		40.74	4.8		38.67	3.9		38.78	2.6		37.41	5.0	
W2	Control	43.69	4.6	0.965 ^A^	45.93	4.2	0.433 ^A^	49.80	2.1	0.839 ^A^	50.29	2.7	0.856 ^A^	47.24	4.4	0.709 ^A^
	Adjuvant	43.16	4.9		43.57	3.2		49.25	4.6		50.08	4.7		46.27	5.2	
	Vaccine	43.66	2.9		46.14	4.6		48.50	3.6		51.17	3.2		47.18	4.4	
W3	Control	49.95	5.2	0.524 ^KW^	51.43	5.7	0.677 ^A^	48.10	3.0	0.535 ^A^	53.29	3.3	0.865 ^A^	50.89	4.6	0.885 ^A^
	Adjuvant	49.33	4.4		49.79	4.9		48.83	3.6		54.08	5.8		50.43	4.9	
	Vaccine	49.28	3.4		52.43	6.1		46.42	4.4		52.58	5.2		50.23	5.2	
W4	Control	53.39	6.1	0.66 ^A^	55.00	4.9	0.408 ^A^	52.50	2.7	0.805 ^A^	48.14	5.5	0.708 ^A^	52.24	5.5	0.499 ^A^
	Adjuvant	53.65	4.9		52.21	4.9		53.17	4.3		46.25	5.9		51.44	5.6	
	Vaccine	55.69	4.2		56.29	7.0		51.50	5.4		48.91	5.6		53.32	6.1	
W5	Control	54.22	6.5	0.622 ^A^	58.00	4.4	0.672 ^A^	56.00	1.7	0.994 ^KW^	52.36	4.2	0.839 ^A^	55.08	4.9	0.736 ^A^
	Adjuvant	54.54	4.1		54.93	5.3		56.25	5.7		51.25	5.6		54.28	5.2	
	Vaccine	52.19	3.2		56.50	8.7		56.00	6.3		50.83	4.6		53.92	6.2	
W6	Control	57.76	7.2	0.660 ^KW^	61.43	4.2	0.52 ^A^	59.10	4.6	0.714 ^A^	54.29	5.9	0.833 ^A^	58.07	6.0	0.465 ^KW^
	Adjuvant	58.01	5.8		58.64	4.8		59.25	7.4		52.83	6.1		57.27	6.2	
	Vaccine	57.31	4.4		57.86	8.3		56.25	8.0		52.33	6.1		56.06	6.8	
W7	Control	59.43	7.6	0.915 ^KW^	60.93	4.8	0.462 ^A^	60.00	5.6	0.488 ^A^	56.79	5.9	0.744 ^A^	59.23	6.0	0.242 ^KW^
	Adjuvant	57.89	6.9		58.14	4.4		59.00	6.1		55.67	7.1		57.7	5.9	
	Vaccine	58.57	5.9		56.93	8.2		55.25	8.4		54.00	6.6		56.31	7.1	
W8	Control	63.01	7.0	0.992 ^A^	66.71	5.3	0.416 ^A^	62.40	3.0	0.433 ^A^	64.36	7.9	0.887 ^A^	64.25	6.1	0.355 ^A^
	Adjuvant	62.57	7.0		61.93	6.6		61.33	6.9		62.67	10		62.13	7.4	
	Vaccine	62.85	6.0		63.36	8.1		58.08	5.9		62.17	6.5		61.73	6.6	
W9	Control	66.21	7.4	0.468 ^KW^	69.86	6.3	0.366 ^A^	66.50	3.8	0.423 ^A^	67.64	9.1	0.705 ^A^	67.63	6.9	0.158 ^A^
	Adjuvant	64.95	7.0		64.64	6.4		64.67	8.0		65.42	9.2		64.91	7.2	
	Vaccine	64.85	7.6		65.64	8.4		60.92	7.9		63.58	7.5		63.86	7.6	

^KW^: Kruskal–Wallis test. ^A^: ANOVA.

## Appendix F

**Table A13:** Average daily gain (ADG) between weighing dates (W) along the experiment in Control, Adjuvant-only, and Vaccine groups in each of the four flocks individually (Flocks 1–4) and all flocks grouped together (All Flocks). Data represented as mean and standard deviation (SD).

	Group	Flock 1			Flock 2			Flock 3			Flock 4			All Flocks		
		*n* = 21			*n* = 21			*n* = 17			*n* = 19			*n* = 78		
		Mean	SD	*p*	Mean	SD	*p*	Mean	SD	*p*	Mean	SD	*p*	Mean	SD	*p*
ADG1 (W2–W1)	Control	273	45	0.982 ^A^	145	78	0.394 ^A^	209	64	0.472 ^A^	216	59	0.898 ^A^	211	76	0.719 ^A^
	Adjuvant	270	44		111	46		203	25		223	43		201	72	
	Vaccine	269	35		102	52		179	35		229	48		194	77	
ADG2 (W3–W2)	Control	116	28	0.763 ^A^	95	38	0.772 ^KW^	−31	90	0.715 ^A^	60	22	0.189 ^A^	67	69	0.227 ^KW^
	Adjuvant	114	23		107	36		−8	65		80	59		76	66	
	Vaccine	104	43		108	78		−39	44		28	54		55	81	
ADG3 (W4–W3)	Control	72	53	0.164 ^KW^	87	46	0.605 ^A^	88	70	0.865 ^A^	−95 ^ab^	57	**0.023** **^A^***	34	96	0.288 ^KW^
	Adjuvant	90	43		59	70		87	39		−145 ^a^	22		27	107	
	Vaccine	134	88		94	82		102	48		−68 ^b^	42		69	102	
ADG4 (W5–W4)	Control	13 ^a^	57	**0.020** **^KW^***	48 ^a^	28	**0.049** **^KW^***	74	35	0.528 ^KW^	84 ^ab^	45	**0.055** **^A#^**	53 ^a^	50	**0.045** **^KW^***
	Adjuvant	14 ^a^	21		43 ^a^	28		66	43		100 ^a^	18		54 ^a^	42	
	Vaccine	−56 ^b^	69		3 ^b^	46		96	43		38 ^b^	54		17^b^	76	
ADG5 (W6–W5)	Control	79	92	0.610 ^KW^	78 ^a^	26	**0.011** **^A^***	69	87	0.270 ^KW^	45	64	0.928 ^A^	67	68	0.146 ^A^
	Adjuvant	77	48		84 ^a^	34		67	62		37	32		67	46	
	Vaccine	114	99		31 ^b^	35		6	69		35	44		48	76	
ADG6 (W7–W6)	Control	43	53	0.205 ^KW^	−13	27	0.827 ^A^	23	61	0.451 ^A^	60	53	0.675 ^A^	29	54	0.384 ^A^
	Adjuvant	−3	57		−13	54		−6	55		67	58		10	61	
	Vaccine	32	102		−23	24		−25	65		40	56		6	71	
ADG7 (W8–W7)	Control	64	32	0.608 ^A^	98	31	0.435 ^KW^	38	66	0.962 ^A^	120	73	0.897 ^A^	83	58	0.589 ^A^
	Adjuvant	84	51		64	66		37	42		111	89		74	65	
	Vaccine	76	21		109	26		45	52		130	28		90	44	
ADG8 (W9–W7)	Control	103	53	0.522 ^KW^	92	80	0.838 ^A^	100	81	0.792 ^KW^	94	76	0.662 ^A^	97	68	0.372 ^A^
	Adjuvant	77	60		80	52		81	38		79	154		79	81	
	Vaccine	65	71		67	99		69	58		40	74		61	74	
**Global** **ADG** **(W9–W1)**	Control	91	15	0.913 ^A^	81 ^a^	14	**0.045** **^A^***	71	11	0.229 ^A^	74	24	0.759 ^KW^	80 ^a^	18	**0.072** **^A#^**
	Adjuvant	89	13		69 ^ab^	11		67	18		70	19		74 ^ab^	17	
	Vaccine	87	23		64 ^b^	11		56	14		63	16		68 ^b^	20	

^A^: ANOVA. ^KW^: Kruskal–Wallis test. ^a,b^: Statistically significant differences between groups based on post hoc test. *: Statistical significance (*p* < 0.05). ^#^: Statistical tendency (*p* ≤ 0.1)

## Appendix G

**Table A14:** Hematological results along the experiment in Control, Adjuvant-only, and Vaccine groups (*n* = 26 each) of all Flocks grouped together. Data represented as mean and standard deviation (SD). H: Hematology date. A reference threshold is provided at the end of the Table.

	Group	WBC ^1^ (×10^3^/mm^3^)		RBC ^2^ (×10^6^/mm^3^)		Hematocrit (%)		Hemoglobin (g/dl)		Platelets (×10^3^/mm^3^)	
		Mean	SD	Mean	SD	Mean	SD	Mean	SD	Mean	SD
H1	Control	7.43	1.88	11.56	0.97	35.52	3.01	11.72	1.04	666	145
	Adjuvant	7.60	2.31	11.65	0.87	35.09	2.95	12.15	0.99	616	183
	Vaccine	7.64	1.86	11.18	0.74	34.53	2.57	11.65	0.69	631	157
H2	Control	7.70	1.89	10.93	0.95	34.29	2.76	11.32	0.93	601	223
	Adjuvant	8.67	2.54	11.14	0.64	34.42	2.80	11.72	0.83	618	233
	Vaccine	7.56	1.31	11.20	0.88	35.21	2.77	11.71	1.00	602	215
H3	Control	7.28	1.48	11.08	1.13	34.96	3.60	11.06	1.22	552	197
	Adjuvant	7.95	2.80	10.96	0.95	34.24	2.93	11.04	1.02	525	198
	Vaccine	7.46	1.98	11.31	0.76	35.68	2.80	11.28	0.78	521	139
H4	Control	8.69	2.03	10.47	0.93	32.49	3.01	10.55	0.95	458	156
	Adjuvant	8.41	1.87	10.61	0.71	32.62	2.18	10.58	0.73	469	146
	Vaccine	8.22	1.62	10.51	0.74	32.51	2.38	10.53	0.77	460	118
H5	Control	7.70	1.46	10.72	0.95	33.50	3.27	10.56	0.75	680	338
	Adjuvant	8.06	1.94	10.80	0.77	33.38	2.76	10.77	0.82	672	379
	Vaccine	7.40	1.56	10.79	0.90	33.59	3.15	10.70	0.90	776	385
H6	Control	6.87	1.70	10.79	1.77	34.64	5.07	10.85	1.58	622	290
	Adjuvant	7.66	2.37	10.97	1.62	34.70	4.93	11.10	1.60	645	398
	Vaccine	6.96	2.06	10.94	1.31	35.11	4.42	11.03	1.14	667	316
H7	Control	7.29	2.50	10.03	1.71	32.58	5.26	10.64	1.62	396	128
	Adjuvant	8.13	2.14	10.40	1.17	33.57	3.60	11.04	1.21	363	242
	Vaccine	6.88	1.52	9.94	1.55	32.33	4.44	10.56	1.35	462	153
H8	Control	7.65	1.90	10.36	1.57	34.40	5.14	11.18	1.29	638	368
	Adjuvant	8.11	1.80	10.67	0.97	35.02	3.38	11.70	0.77	524	292
	Vaccine	7.53	2.19	10.19	1.27	33.70	4.56	11.08	1.11	553	312
H9	Control	8.08	2.31	10.40	1.61	34.72	4.86	11.04	1.68	469	140
	Adjuvant	8.42	2.18	10.57	0.97	34.71	3.71	11.08	1.11	477	210
	Vaccine	8.03	2.39	10.57	1.01	35.02	3.33	11.04	1.01	461	132
H10	Control	8.35	2.71	10.41	1.42	34.95	5.10	10.89	1.36	357	122
	Adjuvant	8.10	1.91	10.45	1.57	34.51	3.97	10.78	1.17	437	191
	Vaccine	8.49	1.98	10.89	1.58	36.10	4.51	11.13	1.22	425	152
H11	Control	9.60	2.51	9.80	1.18	32.67	3.92	10.58	1.31	370	129
	Adjuvant	8.95	1.62	10.14	1.21	33.20	3.43	10.58	1.25	385	134
	Vaccine	9.56	3.00	9.83	0.98	32.49	2.64	10.42	0.90	382	151
H12	Control	7.63	2.18	10.07	1.70	32.97	5.49	10.09	1.76	496	171
	Adjuvant	7.83	1.60	10.48	1.03	33.71	3.28	10.47	1.03	447	192
	Vaccine	7.32	1.23	10.40	0.96	33.69	2.98	10.39	0.94	476	171
**Reference** **Treshold**		**4–12**		**9–14**		**28–40**		**8–15**		**250–750**	

^1^ WBC: White blood cell count. ^2^ RBC: Red blood cell count

## Appendix H

**Table A15:** Histopathological findings in the peripheral nervous system in Control, Adjuvant-only and Vaccine groups of all Flocks grouped together. Data provided as animals with the referred histological lesion relative to the total number of animals analyzed. Methodology of histopathological evaluation is detailed in Table A3, Table A4, Table A5, Table A6, Table A7, Table A8, Table A9, Table A10 and Table A11 (Appendix D).

Location	Group	Perivascular Cuffing	Inflammation
		Presence	Presence
Subcutaneous thoracic nerve	Control	16/24	1/25
	Adjuvant	15/26	1/26
	Vaccine	17/25	2/26
	*p*	0.706 ^Xi^	0.790 ^LR^
Sciatic nerve	Control	12/25	1/26
	Adjuvant	14/26	0/26
	Vaccine	16/26	0/26
	*p*	0.622 ^Xi^	0.320 ^LR^
Tibial nerve	Control	11/26	1/26
	Adjuvant	15/26	1/26
	Vaccine	12/26	1/26
	*p*	0.513 ^Xi^	1000 ^LR^
Radial nerve	Control	15/24	1/24
	Adjuvant	13/26	0/26
	Vaccine	13/23	0/23
	*p*	0.672 ^Xi^	0.324 ^LR^

^Xi^: Pearson’s chi square test. ^LR^: Likelihood ratio test.

## Appendix I

Histopathological results in liver, kidney, spleen, adrenal gland, thyroid gland, and thymus of Control, Adjuvant-only, and Vaccine groups of all Flocks grouped together. Data provided as animals with the referred histological lesion relative to the total number of animals analyzed. Methodology of histopathological evaluation is detailed in Table A3, Table A4, Table A5, Table A6, Table A7, Table A8, Table A9, Table A10 and Table A11 (Appendix D).

**Table A16:** Histopathological findings in the liver.

Location	Group	Portal/Periportal Inflammation			Hepatocytes		
		Presence	Type		Degeneration	Necrosis	Atrophy
			LP ^1^	LP + E ^2^			
Liver	Control	9/26	8/9	1/9	13/26	1/26	14/26
	Adjuvant	12/26	6/12	6/12	15/26	1/26	9/26
	Vaccine	12/26	9/12	3/12	10/26	0/26	12/26
	*p*	0.62 ^LR^	0.13 ^LR^		0.377 ^Xi^	0.439 ^LR^	0.374 ^Xi^

^1^ LP: Lymphoplasmacytic. ^2^ LP + E: Lymphoplasmacytic and eosinophilic. ^LR^: Likelihood ratio test. ^Xi^: Pearson’s chi square test.

**Table A17:** Histopathological findings in the kidney.

Location	Group	Glomeruli	Tubules		Interstitium	Medulla
		Protein	Degeneration	Hyaline Droplets	Inflammation	Mineralization
**Kidney**	Control	15/26	2/26	10/26	8/26	10/26
	Adjuvant	16/26	2/26	9/26	11/26	10/26
	Vaccine	15/26	4/26	12/26	10/26	9/26
	*p*	0.948 ^Xi^	0.589 ^LR^	0.687 ^Xi^	0.681 ^Xi^	0.947 ^Xi^

^Xi^: Pearson’s chi square test. ^LR^: Likelihood ratio test.

**Table A18:** Histopathological findings in the spleen.

Location	Group	White Pulp Hyperplasia	Perifollilular PMs ^1^
**Spleen**	Control	11/26	24/26
	Adjuvant	12/26	25/26
	Vaccine	10/26	23/26
	*p*	0.854 ^Xi^	0.568 ^LR^

^1^ PMs: Polymorphonuclear leukocytes (i.e., neutrophils, eosinophils). ^Xi^: Pearson’s chi square test. ^LR^: Likelihood ratio test.

**Table A19:** Histopathological findings in the adrenal gland.

Location	Group	Cortical Hyperplasia				Inflammation
		Presence	Localization			Presence
			Fascicular	Reticular	Both	
**Adrenal Gland**	Control	13/26	4/12	1/12	7/12	4/26
	Adjuvant	15/26	7/15	3/15	5/15	5/26
	Vaccine	18/26	9/18	1/18	8/18	8/26
	*p*	0.365 ^Xi^	0.558 ^LR^			0.376 ^Xi^

^Xi^: Pearson’s chi square test. ^LR^: Likelihood ratio test.

**Table A20:** Histopathological findings in the thyroid gland.

Location	Group	Inflammation	Follicular Cells Hyperplasia	Follicular Cells Hypertrophy	C Cells Hypertrophy
**Thyroid Gland**	Control	8/26	16/26	0/26	4/26
	Adjuvant	11/26	15/26	3/26	4/26
	Vaccine	4/26	13/26	3/26	7/26
	*p*	0.102 ^Xi^	0.694 ^Xi^	**0.078 ^LR#^**	0.489 ^LR^

^Xi^: Pearson’s chi square test. ^LR^: Likelihood ratio test. ^#^: Statistical tendency (*p* ≤ 0.1).

**Table A21:** Histopathological findings in the thymus.

Location	Group	Germinal Centers	Degree of Involution			
			0	1	2	3
**Thymus**	Control	4/26	13/25	10/25	0/25	2/25
	Adjuvant	1/26	9/26	16/26	0/26	1/26
	Vaccine	0/26	11/26	11/26	0/26	4/26
	*p*	**0.043 ^LR^***	0.364 ^LR^			

^LR^: Likelihood ratio test. *: Statistical significance (*p* < 0.05).

## References

[1] Greenwood B. (2014). The contribution of vaccination to global health: Past, present and future. Philos. Trans. R. Soc. B Biol. Sci..

[2] Lacasta D., Ferrer L.M., Ramos J.J., González J.M., Ortín A., Fthenakis G.C. (2015). Vaccination schedules in small ruminant farms. Vet. Microbiol..

[3] Morens D.M., Folkers G.K., Fauci A.S. (2004). The challenge of emerging and re-emerging infectious diseases. Nature.

[4] UE Commission Decision 2008/655/EC, 24 July 2008, Approving the Emergency Vaccination Plans Against Bluetongue of Certain Member States and Fixing the Level of the Community’s Financial Contribution for 2007 and 2008. https://eur-lex.europa.eu/legal-content/EN/TXT/PDF/?uri=CELEX:32008D0655&from=EN.

[5] Mellor P.S., Carpenter S., Harrup L., Baylis M., Mertens P.P.C. (2008). Bluetongue in Europe and the Mediterranean Basin: History of occurrence prior to 2006. Prev. Vet. Med..

[6] Agence Française de Sécurité Sanitaire (AFSS) Review of Adverse Affects Observed after Vaccination Against Bluetongue, Serotype 1 and Serotype 8, as of 31/05/2009. https://www.anses.fr/en/system/files/ANMV-Fi-VaccinFCOEN.pdf.

[7] Nusinovici S., Seegers H., Joly A., Beaudeau F., Fourichon C. (2011). A side effect of decreased fertility associated with vaccination against bluetongue virus serotype 8 in Holstein dairy cows. Prev. Vet. Med..

[8] Dyer F., Brown E., Cooles S., Tait A. (2009). Suspected adverse reactions, 2008. Vet. Rec..

[9] González J.M., Figueras L., Ortega M.E., Lozano M., Ruiz De Arcaute M., Royo R., Cebrián L.M., Ferrer L.M., Fariñas F., De Jalón J.A.G. (2010). Possible adverse reactions in sheep after vaccination with inactivated BTV vaccines. Vet. Rec..

[10] Asín J., Pérez M., Pinczowski P., Gimeno M., Luján L. (2018). From the bluetongue vaccination campaigns in sheep to overimmunization and ovine ASIA syndrome. Immunol. Res..

[11] Luján L., Pérez M., Salazar E., Álvarez N., Gimeno M., Pinczowski P., Irusta S., Santamaría J., Insausti N., Cortés Y. (2013). Autoimmune/autoinflammatory syndrome induced by adjuvants (ASIA syndrome) in commercial sheep. Immunol. Res..

[12] Shoenfeld Y., Agmon-Levin N. (2011). “ASIA”—Autoimmune/inflammatory syndrome induced by adjuvants. J. Autoimmun..

[13] Burakova Y., Madera R., McVey S., Schlup J.R., Shi J. (2017). Adjuvants for Animal Vaccines. Viral Immunol..

[14] Shardlow E., Mold M., Exley C. (2018). Unraveling the enigma: Elucidating the relationship between the physicochemical properties of aluminium-based adjuvants and their immunological mechanisms of action. Allergy Asthma Clin. Immunol..

[15] Asín J., Molín J., Pérez M., Pinczowski P., Gimeno M., Navascués N., Muniesa A., de Blas I., Lacasta D., Fernández A. (2019). Granulomas Following Subcutaneous Injection With Aluminum Adjuvant-Containing Products in Sheep. Vet. Pathol..

[16] de Miguel R., Asín J., Rodríguez-Largo A., Molín J., Echeverría I., de Andrés D., Pérez M., de Blas I., Mold M., Reina R. (2020). Detection of aluminum in lumbar spinal cord of sheep subcutaneously inoculated with aluminum-hydroxide containing products. J. Inorg. Biochem..

[17] Asín J., Pascual-Alonso M., Pinczowski P., Gimeno M., Pérez M., Muniesa A., de Pablo-Maiso L., de Blas I., Lacasta D., Fernández A. (2020). Cognition and behavior in sheep repetitively inoculated with aluminum adjuvant-containing vaccines or aluminum adjuvant only. J. Inorg. Biochem..

[18] Vandevelde M., Higgins R.J., Oevermann A. (2012). Veterinary Neuropathology: Essentials of Theory and Practice.

[19] Cerviño M., Figueras L., Martín S., Elvira L., Callus M., Dowlut S., Engelhard I., Calvo E., Makoschey B. (2011). Specific humoral response and effect on rectal temperature of two clostridial vaccines in lambs. Vet. Rec..

[20] Troxel T.R., Gadberry M.S., Wallace W.T., Kreider D.L., Shockey J.D., Colburn E.A., Widel P., Nicholson I. (2001). Clostridial antibody response from injection-site lesions in beef cattle, long-term response to single or multiple doses, and response in newborn beef calves. J. Anim. Sci..

[21] Silva G.M., Poore M.H., Ranches J., Moriel P. (2018). Effects of timing of vaccination relative to weaning and post-weaning frequency of energy supplementation on growth and immunity of beef calves1. J. Anim. Sci..

[22] Arthington J.D., Cooke R.F., Maddock T.D., Araujo D.B., Moriel P., Dilorenzo N., Lamb G.C. (2013). Effects of vaccination on the acute-phase protein response and measures of performance in growing beef calves1. J. Anim. Sci..

[23] Moriel P., Arthington J.D. (2013). Metabolizable protein supply modulated the acute-phase response following vaccination of beef steers. J. Anim. Sci..

[24] Reeds P.J., Jahoor F. (2001). The amino acid requirements of disease. Clin. Nutr..

[25] Arts R.J.W., Carvalho A., La Rocca C., Palma C., Rodrigues F., Silvestre R., Kleinnijenhuis J., Lachmandas E., Gonçalves L.G., Belinha A. (2016). Immunometabolic Pathways in BCG-Induced Trained Immunity. Cell Rep..

[26] O’Neill L.A.J., Kishton R.J., Rathmell J. (2016). A guide to immunometabolism for immunologists. Nat. Rev. Immunol..

[27] Macías-Cruz U., Stevens J.C., Correa-Calderón A., Mellado M., Meza-Herrera C.A., Avendaño-Reyes L. (2018). Effects of pre-lambing maternal energy supplementation on post-weaning productive performance and thermoregulatory capacity of heat-stressed male lambs. J. Therm. Biol..

[28] Al-Dawood A. (2017). Towards heat stress management in small Ruminants—A review. Ann. Anim. Sci..

[29] Varela-Martínez E., Abendaño N., Asín J., Sistiaga-Poveda M., Pérez M.M., Reina R., de Andrés D., Luján L., Jugo B.M. (2018). Molecular Signature of Aluminum Hydroxide Adjuvant in Ovine PBMCs by Integrated mRNA and microRNA Transcriptome Sequencing. Front. Immunol..

[30] Henry B.A., Pope M., Birtwistle M., Loughnan R., Alagal R., Fuller-Jackson J.P., Perry V., Budge H., Clarke I.J., Symonds M.E. (2017). Ontogeny and thermogenic role for sternal fat in female sheep. Endocrinology.

[31] Kherani Z.S., Auer R.N. (2008). Pharmacologic analysis of the mechanism of dark neuron production in cerebral cortex. Acta Neuropathol..

[32] Zimatkin S.M., Bon E.I. (2018). Dark Neurons of the Brain. Neurosci. Behav. Physiol..

[33] Garman R.H. (2011). Histology of the Central Nervous System. Toxicol. Pathol..

[34] Jortner B.S. (2006). The return of the dark neuron. A histological artifact complicating contemporary neurotoxicologic evaluation. Neurotoxicology.

[35] Varela-Martínez E., Bilbao-Arribas M., Abendaño N., Asín J., Pérez M., de Andrés D., Luján L., Jugo B.M. (2020). Whole transcriptome approach to evaluate the effect of aluminium hydroxide in ovine encephalon. Sci. Rep..

[36] Goto N., Ueno G., Iwasa S. (1987). Swelling Reaction of the Pancreas in Guinea Pigs Caused by Aluminum-Adsorbed Diphtheria-Purified Pertussis-Tetanus Combined Vaccine. Microbiol. Immunol..

[37] AEMET OpenData—State Meteorological Agency—AEMET—Spanish Government. http://www.aemet.es/en/datos_abiertos/AEMET_OpenData.

